# Prevention of Ageing—The Role of Micro-Needling in Neck and Cleavage Rejuvenation: A Narrative Review

**DOI:** 10.3390/ijerph19159055

**Published:** 2022-07-25

**Authors:** Justyna Pająk, Jacek C. Szepietowski, Danuta Nowicka

**Affiliations:** 1 Department of Dermatology, Venereology and Allergology, Wrocław Medical University, 50-368 Wroclaw, Poland; jpajakowna@gmail.com (J.P.); jacek.szepietowski@umw.edu.pl (J.C.S.); 2 Faculty of Physiotherapy, Wroclaw University of Health and Sport Sciences, 51-612 Wroclaw, Poland

**Keywords:** micro-needling, skin ageing, aesthetic medicine, skin remodelling

## Abstract

Although interest in aesthetic medicine is growing, the focus is often placed outside of the facial area, namely on the skin of the neck and cleavage. Exposure to the sun and muscle movements cause the prompt development of wrinkles that may appear there, even before they show up on the face. We conducted a literature review devoted to micro-needling to identify its role in anti-ageing treatments and to determine the gaps in current knowledge. A search in Medline identified 52 publications for neck and face micro-needling. Micro-needling is an anti-ageing procedure that involves making micro-punctures in the skin to induce skin remodelling by stimulating the fibroblasts responsible for collagen and elastin production. It can be applied to the skin of the face, neck, and cleavage. Two to four weeks should be allowed between repeated procedures to achieve an optimal effect. The increase in collagen and elastin in the skin can reach 400% after 6 months, with an increase in the thickness of the stratum granulosum occurring for up to 1 year. In conclusion, micro-needling can be considered an effective and safe aesthetic medicine procedure which is conducted at low costs due to its low invasiveness, low number of adverse reactions, and short recovery time. Little evidence identified in the literature suggests that this procedure requires further research.

## 1. Introduction

Nowadays, not only is increasing life expectancy important, but so is improving people’s quality of life, which involves maintaining a youthful appearance and slowing the ageing of the skin. Physical attractiveness is a serious matter in society, with youthfulness being one of its components [1]. Surpassing others in appearance and physical beauty brings benefits to individuals in multiple domains, resulting in multidimensional consequences [2,3]. This is why aesthetic medicine is becoming more and more popular [4]. Until recently, the primary area of interest was facial skin, but now, it focuses also on other representative parts of the body such as neck and cleavage skin. Being particularly vulnerable to ageing factors, mainly sun exposure and muscle movements, wrinkles may appear here, even before they show up on the face. Sun exposure leads to increased transepidermal water loss and sebum level, as well as a reduced water content in the stratum corneum [5]. This results in increasing demand for rejuvenating treatments in that area. Hyaluronic acid, laser therapy and chemical peeling are commonly used; however, thanks to its low invasiveness and wider availability, mesotherapy, especially micro-needling, is chosen more and more often. This medical procedure helps to prevent skin ageing by creating micro-punctures in it and stimulating its remodelling, resulting in the reduction of wrinkles, discolouration, and stretch marks. The procedure is also called collagen-induction therapy or percutaneous collagen induction. It has been proven that the treatment of certain skin defects results in the improved quality of patients’ lives [6].

Reports on micro-needling from the literature focus mainly on face skin [7,8,9,10,11] and there are still very few that discuss its effect on the neck and cleavage [12,13]. Furthermore, micro-needling is often used in combination with other treatments, which makes an assessment of its efficacy and safety more challenging. The aim of this article was to conduct a review of the literature on micro-needling in order to identify its role in anti-ageing treatments, determine the gaps in the current knowledge, and indicate directions for further research in that area.

## 2. Ageing of the Skin

Ageing of the skin is a natural process. To describe this process, the term “exposome” has been introduced, which takes into account, in addition to widely discussed external and internal factors, the response of the human body [14]. Genetics is considered an internal factor. A comparable level of ageing takes place in almost every other tissue of the human body and is similar across the population, except for those with rare genetic diseases [15]. However, in contrast to this, external factors affecting the skin depend highly on individual lifestyles. According to Krutmann et al., environmental factors include sun radiation (ultraviolet (UV) radiation, visible light, infrared radiation), air pollution, tobacco smoke, nutrition, cosmetic products, and other factors [14].

The epidermis consists of keratinocytes (95% of all cells), melanocytes, Langerhans cells, and Merkel cells [15]. In young skin, keratinocytes are regular and polygonal with surrounding thin reticulated collagen fibres. Over time, the shape of keratinocytes becomes irregular with the development of unevenly distributed pigmentation. Next, in the elderly, keratinocytes are seen with marked alterations, while collagen tends to form a cluster of curled fibres typical of elastosis [16]. Melanocytes located in the bottom layer (the stratum basale) of the epidermis produce melanin, which creates a shell around the nuclei of the surrounding keratinocytes and protects them against UV radiation [17]. The density of melanocytes decreases approximately 6–8% every 10 years, with a greater impact on skin not exposed to the sun, resulting in pigmentation disorders causing clinically significant discolouration [15]. Langerhans cells are present in all layers of the epidermis, mainly in the stratum spinosum. Being tissue-resident macrophages of the skin, they are involved in immune homeostasis. Studies have shown that due to the decrease in the population of Langerhans cells with age, the risk of skin cancer among elderly people increases [18]. Merkel cells are primarily found in the stratum basale (similarly to melanocytes) and are considered to be a part of the dispersed neuroendocrine system—they are essential mechanoreceptors for light touch-sensation and produce a variety of neuropeptides (such as a vasoactive intestinal peptide, calcitonin gene-related peptide, substance P, met-enkephalin, and somatostatin) [19,20]. Ageing and sun exposure also lead to an increase in the number of Merkel cell carcinomas [21]. The sebaceous glands located in the skin secrete sebum with moisturizing and oiling features and protect the skin from drying. Sebum production is especially increased during infancy and puberty. With age, the sebaceous glands enlarge and cause widening of the skin pores, with a decrease in sebum secretion by about 20% to 30% every 10 years (which also occurs because of androgen reduction) [15]. It leads to progressive reduction in ceramide production, with a negative effect on the ability of the skin to retain water and lipids in the epidermis, and subsequent development of areas of skin exfoliation due to its dryness [22].

Between the epidermis and the dermis, there is a dermal–epidermal junction, going from the top. It consists of basal keratinocytes with their membranes attached to the lower tissue by hemidesmosomes, lamina lucida, lamina dens, and lamina reticularis. The junction plays a crucial role in transport, protection, and support for the dermis. Ageing processes cause thinning and flattening of the junction, leading to problems with the transport of nutrients between the epidermis and dermis. With the flattening of the junction, the thickness of the epidermis and its cell-turnover time decrease, resulting in slower wound healing and increased skin vulnerability to damage [15,23].

The two-layer dermis consists of the papillary dermis and reticular dermis and is made up of collagen fibres (90% of type I and type III collagen), elastic fibres, blood vessels, and nerve endings. Collagen fibres provide tensile strength and compression resistance for the whole tissue. With age, fibroblasts present in the dermis become less active, leading to a decline in collagen production and in its quality (the diameter of collagen fibres becomes reduced) and an increase in metalloproteinase production [24]. They lead to increased degradation of collagen, particularly type I collagen. Lower activation of fibroblasts also causes a decline in glycosaminoglycans, including hyaluronic acid, which play a role in maintaining proper hydration of the skin. In addition, the subpopulation of fibroblasts—myofibroblasts—decreases with age, which impedes the wound-healing process [15]. Elastic fibres in the dermis are responsible for maintaining the primary shape of the skin [24]. As we age, elastase concentration in the skin increases, causing decomposition of elastic fibres and loss of skin firmness. Biomechanical changes translate into the loss of resilience and elasticity, mainly due to chronic sun exposure [25]. It is also pronounced by the progressive calcification of elastic fibres and their breakdown. Photoageing leads to the accumulation of elastin-breakdown products in the papillary dermis. The process is called elastosis and is clinically manifested as papules, nodules, and deep furrows [26]. Subcutaneous tissue is built of adipocytes creating a protective layer against mechanical and thermal factors; its thinning with age weakens this barrier and negatively affects the appearance of the skin. Regarding differences between skin types, the water content on the stratum corneum and transepidermal water loss level are similar across different skin types in mature skin; however, oily skin is characterised by a higher content of sebum and thicker epidermis [27].

All of the above-described ageing processes within each skin layer result in a characteristic clinical picture, including skin dryness and laxity, wrinkles, discolouration, telangiectasia, and widened skin pores [15,28]. This plethora of processes gives numerous points of grip for treatment procedures. Knowing them creates opportunities to control and slow ageing processes by choosing the appropriate methods.

## 3. Micro-Needling Technique

Micro-needling is one of the anti-ageing procedures that has become increasingly popular. The procedure involves making micro-punctures in the skin, preferably in series, to achieve an even result on the entire surface. It can be performed using a dermo-roller (a device of the simplest design comprising a special roll with 192 micro-needles with a diameter of 0.1 mm and an average length of 1.5 mm), a derma-stamp (a miniature version of a dermo-roller used for smaller areas), or a derma-pen (an automatic device with an adjustable length of the needle up to 3 mm and operating frequency according to the type of skin surface) [13]. The depth of the micro-punctures is very important for the success of the procedure and for obtaining appropriate results, and depends on the area of the skin (different epidermis thickness) and individual features. Usually, the proper depth is established by observing so-called “pinpoint bleeding” [29,30]. That depth on the neck and cleavage skin is estimated to be between 1.5 and 3 mm [30]. Such punctures may be painful, so topical analgesic creams can be used. Local anaesthesia is usually obtained with lidocaine in a gel or cream, which significantly reduces pain. However, it has been proven that lidocaine and mepivacaine can impede the healing process, so if the patient withstands the whole procedure without anaesthesia, it is the most effective way to proceed in light of recent research [31]. The correctly performed procedure involves a proper technique for the punctures themselves; first, within the area of the skin, perpendicular movements should be made, then, parallel, and at the end, oblique ones from one side to the other. Patients are less anxious and stressed if micro-needles are used rather than normal-sized needles [32]. The technique is not difficult to master.

## 4. Effects of Micro-Needling

Many authors claim that micro-punctures induce skin remodelling by stimulating the fibroblasts responsible for collagen and elastin production [29]. There are three stages in this process: inflammation, proliferation, and remodelling. Obtaining punctate haemorrhages due to skin blood-vessel punctures leads to the release of platelets and numerous growth factors such as platelet-derived growth factor, transforming growth factor-alpha, and epidermal growth factor [29,30,33]. The punctures immediately stimulate fibroblast activity, which starts the one-week stage of inflammation [29]. The next phase (proliferation) is dependent on the growth factors mentioned above, metalloproteinases, and interleukins. Fibroblasts and keratinocytes proliferate and produce collagen (mostly type I, III, IV, and VII collagen). The structure of collagen fibres is similar to that of undamaged skin [8]. Simultaneously, new blood vessels are created that provide adequate vascularization and oxygenation for the newly formed tissue. During the last phase (remodelling), the epidermis becomes thicker (the number of stratum granulosum cells increases) and further, collagen production takes place (mostly type III collagen), which is then transformed to type I collagen that is responsible for the strength and flexibility of the skin. Reversing effects of photoageing have also been observed; proper and regular alignment of the elastin fibres was restored, resulting in a reduction in wrinkles and improvement in skin tension [30]. In general, micro-punctures performed on patients’ skin launch a cascade of regeneration processes similar on a micro-scale to the natural healing process. To maintain the treatment effects and provide time for all the described stages, it is recommended to wait 2–4 weeks between micro-needling sessions [30,34] or even up to 8 weeks, according to some authors [33]. For best results, several micro-needling sessions should be performed.. In the 2008 research, it was proven that after repeated micro-needling procedures, the amount of collagen and elastin in the skin of the patients increased by 400% after 6 months and the stratum granulosum became noticeably thicker after 1 year [8,33,35]. Post-treatment recommendations should include the avoidance of sun exposure and the use of photoprotective topical cream and skin-moisturizing non-allergenic cream for around one week, as well as the maintenance of basic hygiene [30].

## 5. Safety of Micro-Needling

Although many types of research have proved that micro-needling is a safe and minimally invasive medical procedure [8,29,30,35,36], possible adverse reactions can occur. They include transient erythema due to vessel dilation, oedema, increased water loss through the skin, and superficial epidermis exfoliation, while skin infections, contact allergy to needles, and the formation of granulomas are less frequently reported [30,33,34,36]. To reduce the likelihood of adverse reactions, the procedure should not be performed on patients with active skin infections, exacerbation of acne, active herpes labialis, or any other skin lesions in the planned area of treatment [30,33]. A tendency to form keloids, the occurrence of a chronic skin disease with a positive Köbner sign (i.e., psoriasis, vitiligo, lichen planus), autoimmune diseases, and immunosuppression are contradictions to micro-needling. The occurrence of the above-mentioned conditions can worsen their course, reduce the effectiveness of micro-needling, or make its effect unpredictable [30,37]. There is no consensus on using anticoagulants. Aashim et al. mention them as a contraindication [33], but Tina et al. do not recognize them as such [30]. Micro-needling should be performed with particular care in patients infected with human deficiency virus, and hepatitis virus B and C for epidemiological reasons, and in patients who have undergone other aesthetic medicine treatments in the given skin area. This particularly applies to the botulinum toxin and the risk of its spillage [30]. It is recommended to create full documentation of the procedure, both medical and photographic.

Despite the high potential of micro-needling itself, some researchers try to increase the effectiveness of the treatment by adding active substances to the procedure such as vitamin A and C, platelet-rich plasma, and hyaluronic acid [33]. Micro punctures created by micro-needling may improve the penetration of there substances into deeper layers of the skin during the procedure, right after micro-needling or up to 72 h afterwards [29,38]. Active substances can possibly penetrate the stratum spinosum more easily and reach the dermis. Research by Serrano et al. also showed a widening of the funnels of hair follicles by 47%, which additionally facilitates penetration [33,38]. Hair follicles full of dead skin cells or sebum are also cleaned by micro-needling [38].

## 6. Discussion

Skin ageing on the neck and cleavage has become a problem increasingly reported by female patients. Until recently, the facial skin was at the centre of aesthetic medicine attention; however, when it is well-groomed, it is the condition of the skin on the neck, cleavage, or hands that may reveal the true age of the patient. These areas share an environment similar to the facial skin, with exposure to external factors, and are visible to others. This causes psychological discomfort among women whose skin on the cleavage or neck has significantly aged, showing wrinkles, discolouration, stretch marks (atrophic scars) or excessive laxity and dryness. On the neck, there may be a loss of lower-jaw contour, widening of the cervicomental angle, accumulation of fat deposits around the chin, and a loss of neck muscle volume [39]. Wrinkles on the neck and cleavage appear due to upper limb movements and strain caused by having breasts. Practising sport, frequent use of the pectoral girdle muscles, and side-sleeping can also make them deeper. Excessive sun exposure is another ageing factor. UVA radiation constitutes 95% of all UV radiation reaching the Earth and is the main cause of photoageing. It is not blocked by clouds or clear glass and, passing through the epidermis, it causes damage underneath the dermis. Photoageing occurs faster if there are vitamin deficiencies; this mostly includes vitamin A, but also C and E. They have antioxidant properties and protect skin cells from free-radical damage [40]. Other visible effects of photoageing include pigmentation disorders and skin cancer development. Pigmentation disorders manifest themselves as discolouration, and solar and senile lentigines. The last two can turn into pre-cancerous lesions such as actinic keratosis or cancer such as squamous cell carcinoma and melanoma. Treatment of them can result in additional cosmetic defects due to scars after excision. During rapid growth or weight gain, stretch marks can appear on the cleavage. They are usually located near the breasts, and are radiant or perpendicular towards the nipples.

## 7. Materials and Methods

**Figure 1:**
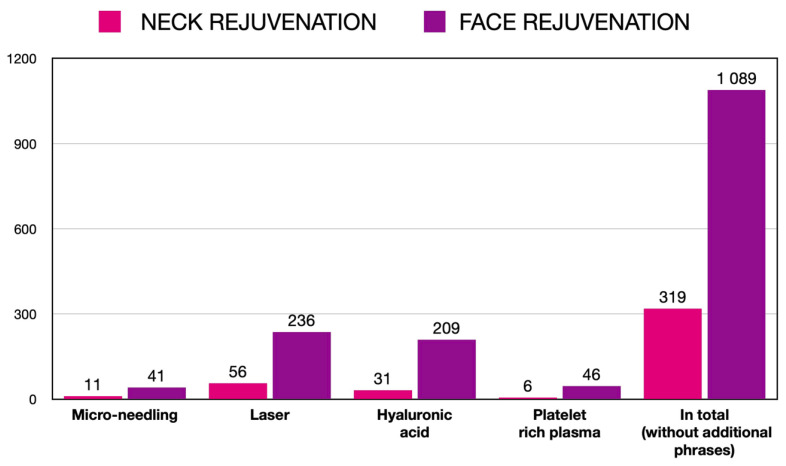
Number of PubMed hits for micro-needling, laser, hyaluronic acid, and platelet-rich plasma by the area of rejuvenation.

The neck and cleavage areas are not common objects of research studies. For the purpose of this review, the Medline database via PubMed was searched for publications on micro-needling and other aesthetic medicine procedures performed on the neck and cleavage. Using the phrase “neck rejuvenation” and limiting the publication period to 2015–2020, there were 319 results found, compared to 1089 results on “face rejuvenation” phrase. Those findings were then separately searched again using “micro-needling”, “laser”, “hyaluronic acid”, and “platelet rich plasma” as additional phrases. The distribution of the results is depicted in Figure 1. Publications on micro-needling constitute around 3% of the results of both searches, which is much less in comparison to publications on laser, hyaluronic acid and platelet-rich plasma. This shows the need for further research in that area.

## 8. Conclusions

Micro-needling can be considered an effective and safe aesthetic medicine procedure which is conducted at low costs due to its low invasiveness, low number of adverse reactions, and short recovery time. It can be used as a single procedure or in combination with other treatments, with additional benefits of repeated treatments. Nevertheless, little evidence identified in the literature suggests that this procedure requires further research.

## Data Availability

Not applicable.
